# A global examination of ecological niche modeling to predict emerging infectious diseases: a systematic review

**DOI:** 10.3389/fpubh.2023.1244084

**Published:** 2023-11-02

**Authors:** Ted J. Lawrence, Bryce P. Takenaka, Aastha Garg, Donghua Tao, Sharon L. Deem, Eric M. Fèvre, Ilona Gluecks, Vasit Sagan, Enbal Shacham

**Affiliations:** ^1^Taylor Geospatial Institute, St. Louis, MO, United States; ^2^College for Public Health and Social Justice, Saint Louis University, St. Louis, MO, United States; ^3^Medical Center Library, Saint Louis University, St. Louis, MO, United States; ^4^Institute for Conservation Medicine, Saint Louis Zoo, St. Louis, MO, United States; ^5^International Livestock Research Institute, Nairobi, Kenya; ^6^Institute of Infection, Veterinary and Ecological Sciences, University of Liverpool, Liverpool, United Kingdom; ^7^Department of Earth and Atmospheric Sciences, Saint Louis University, St. Louis, MO, United States

**Keywords:** disease early detection, ecological and environmental phenomena, epidemiology, One Health, outbreaks, public health

## Abstract

**Introduction:**

As emerging infectious diseases (EIDs) increase, examining the underlying social and environmental conditions that drive EIDs is urgently needed. Ecological niche modeling (ENM) is increasingly employed to predict disease emergence based on the spatial distribution of biotic conditions and interactions, abiotic conditions, and the mobility or dispersal of vector-host species, as well as social factors that modify the host species’ spatial distribution. Still, ENM applied to EIDs is relatively new with varying algorithms and data types. We conducted a systematic review (PROSPERO: CRD42021251968) with the research question: *What is the state of the science and practice of estimating ecological niches* via *ENM to predict the emergence and spread of vector-borne and/or zoonotic diseases?*

**Methods:**

We searched five research databases and eight widely recognized One Health journals between 1995 and 2020. We screened 383 articles at the abstract level (included if study involved vector-borne or zoonotic disease and applied ENM) and 237 articles at the full-text level (included if study described ENM features and modeling processes). Our objectives were to: (1) describe the growth and distribution of studies across the types of infectious diseases, scientific fields, and geographic regions; (2) evaluate the likely effectiveness of the studies to represent ecological niches based on the biotic, abiotic, and mobility framework; (3) explain some potential pitfalls of ENM algorithms and techniques; and (4) provide specific recommendation for future studies on the analysis of ecological niches to predict EIDs.

**Results:**

We show that 99% of studies included mobility factors, 90% modeled abiotic factors with more than half in tropical climate zones, 54% modeled biotic conditions and interactions. Of the 121 studies, 7% include only biotic and mobility factors, 45% include only abiotic and mobility factors, and 45% fully integrated the biotic, abiotic, and mobility data. Only 13% of studies included modifying social factors such as land use. A majority of studies (77%) used well-recognized ENM algorithms (MaxEnt and GARP) and model selection procedures. Most studies (90%) reported model validation procedures, but only 7% reported uncertainty analysis.

**Discussion:**

Our findings bolster ENM to predict EIDs that can help inform the prevention of outbreaks and future epidemics.

**Systematic review registration:**

https://www.crd.york.ac.uk/prospero/, identifier (CRD42021251968).

## 1. Introduction

Amid an increasing global trend in emerging infectious diseases (EIDs) and outbreaks (1, 2), the need to better understand underlying drivers of EIDs has never been greater (3). The social and environmental conditions and their complex interactions that have been commonly implicated as driving EIDs include: climate and land-use change; human-wildlife interactions; shifting human demographics and behaviors including livelihood patterns; and poverty and inequality (1, 4–6). However, modeling the fundamental mechanisms that drive EIDs is one of the most complex and challenging scientific problems as these factors are intricately intertwined and difficult to disentangle (7–10). Thus, there has been hesitance in traditional epidemiology to model these outcomes as the predictive accuracy is questionable (8).

**Figure 1:**
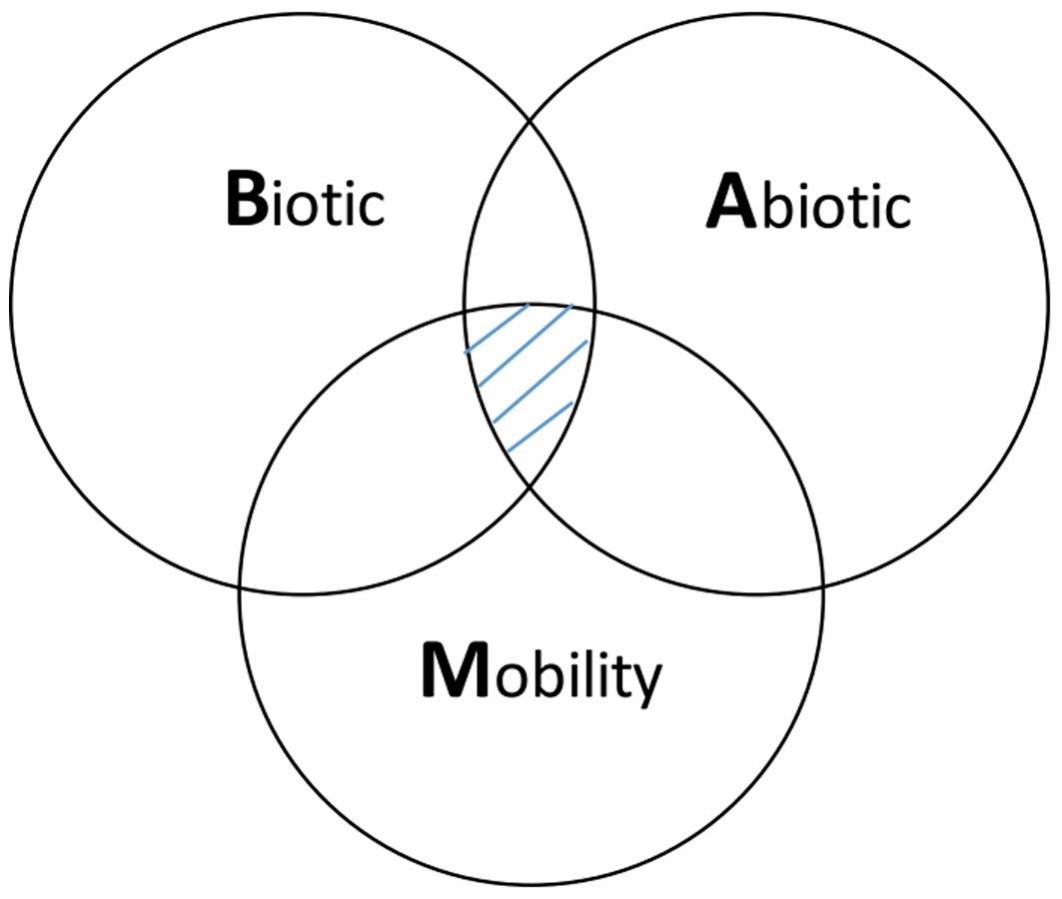
Biotic-abiotic-mobility (BAM) framework. The BAM framework is a simplified representation of a species’ geographic distribution determined by the intersection of suitable biotic conditions and interactions, abiotic conditions, and the species’ mobility, which is shown in the pinstriped center of the diagram. The abiotic component refers to the physical environment (e.g., temperature, precipitation, soil type) that limits the presence of a host-pathogen. The biotic component refers to the biological environment that shapes the distribution of a host-pathogen. Biotic factors include the biological components present in the environment (i.e., biotic conditions), such as vegetation and land cover and the biotic interactions between hosts and vectors, such as vector, host density and distribution that can promote or limit a pathogen’s occurrence. Mobility or dispersal capacity of vector-host species refers to the areas that are accessible to a vector-host species due to limited dispersal abilities or biogeographical barriers.

Ecological niche modeling (ENM) is an approach that expands beyond traditional spatial epidemiology. Specifically, ENM is intended to determine geographic distribution and spatial relationships of environmental conditions, such as climate and land cover with disease occurrence, and thereby represent the ecological niche of the disease (11, 12). ENM enables the spatial prediction and mapping of geographic regions (beyond the locations analyzed) that are at risk to EIDs, so that public health measures can be implemented to prevent future outbreaks and epidemics. The main elements to ENM include occurrence data, environmental data (abiotic and/or biotic variables), and correlative or classification algorithms that link environmental conditions and disease occurrence (11). Ecological niches comprise environmental spaces that include sets of ecological variables, such as precipitation, land cover, and soil characteristics as well as human social factors that modify the behavior and spatial distribution of a host species, such as land use and types of land tenure that influence habitat modifications. These factors shape the potential occurrence of pathogens; and these niches translate into geographic distributions according to the combined effects of the spatial structure of the biotic conditions and interactions, abiotic conditions, and mobility or dispersal capacity of vector-host species (Figure 1) (12, 13).

Elucidating ecological niches in the ENM process is primarily based on algorithms that can be divided into three categories: presence-absence; presence-background; and presence-only (11). Presence is defined as the number of observed occurrences or presence sites of each individual from a host species. Presence-absence algorithms compare environmental conditions where a pathogen is present versus where it is absent. The presence-absence approach can be highly robust, but absence data may be limited due to actual absence, lack of data or untested data, and many datasets may only contain locations of presence. Such limited datasets of absences are due to incomplete sampling efforts needed to be confident of absence, which is often difficult to achieve and estimates of species range changes based on survey data (14). Presence-background algorithms involve the same algorithms used for presence-absence models, but include *background samples*, which are usually based on random sampling of locations taken where the presence or absence of a species is unknown in the study area (15–17). The sampled background points are then used as pseudo-absences (18). The background points are compared to the observed presence data to help differentiate the environmental conditions under which a species can occur or not (15). The presence-background algorithms are sometimes referred to as enhanced presence-only algorithms and are intended to give a more accurate species distribution prediction than simple presence-only algorithms (19). Presence-only algorithms focus solely on the environmental values linked to each occurrence record for calibration (i.e., no direct information about absences is used) (20). Each of these model types enable the spatial prediction of EIDs, while the choice of approach is largely dependent on scale of analysis and data availability.

In the early 2000s, professionals concerned with multi-host pathogens began applying ENM to disease transmission systems (21). Since then, ENM applied to EIDs has been increasingly employed to model diverse pathogens with a focus on emerging and re-emerging vector-borne and zoonotic diseases (22). However, ENM applied to EIDs has been criticized due to the limited understanding of users regarding the potential data and algorithm limitations of this modeling framework (11). This is especially true for the inclusion of all BAM factors (Figure 1), which is considered the most rigorous approach and the users understanding of their biological meaning, assumptions, and available data in ENM (22). Moreover, few practical guidelines exist on the proper application of both BAM and ENM techniques (23) and especially applied to EIDs. Due to the growing, yet relatively new and limited number of studies using this approach, a better understanding about the application of ENM to EIDs is needed.

We conducted a global examination (via systematic review) of ENM to predict EIDs as a response to the need to better understand such applications. Our research question was: *What is the state of the science and practice of estimating ecological niches* via *ENM to predict the emergence and spread of vector-borne and/or zoonotic diseases?* We focused our research question on vector-borne and zoonotic diseases because the majority of emerging infectious diseases are zoonotic (24) and vector-borne diseases are major causes of mortality and morbidity globally (5, 25). Our objectives for this study were to: (1) describe the growth and distribution of studies across the types of infectious diseases, scientific fields, and geographic regions; (2) evaluate the likely effectiveness of the studies to represent ecological niches based on the BAM framework; (3) explain some potential pitfalls of ENM algorithms and techniques used in those studies; and (4) provide specific recommendations for future studies on the analysis of ecological niches to predict EIDs. Our intent is not to provide a practical step-by-step guide to the application of ENM, but rather to provide an overview of the science and practice of ENM that can bolster collaborations between non-experts and ENM experts on interdisciplinary teams to predict infectious disease outbreaks and epidemics. Our findings and recommendations can inform professionals concerned with multi-host pathogens that focus on the interconnections between the health of humans, animals, and their shared environments to help prevent outbreaks and future epidemics (26).

## 2. Methods

### 2.1. Literature search

Our systematic review followed PRISMA (Preferred Reporting Items for Systematic Reviews and Meta-analyses) guidelines (27) and was registered as a protocol with PROSPERO (registration number: CRD42021251968). The bibliographic search of scientific articles was performed using Scopus, ISI Web of Science, PubMed, Medline, and Google Scholar databases. Also, we searched widely recognized One Health journals that publish articles on the linkages between environmental factors and EIDs, which included: EcoHealth; One Health; International Journal of One Health; One Health Outlook; Infection Ecology and Epidemiology; The Lancet Planetary Health; Challenges; and Frontiers in Public Health – Planetary Health. Our search terms were based on the concepts of modeling geospatial relationships between environmental factors and EIDs, and the scientific fields that conduct such research (Appendix 1). We did a pairwise search involving each individual search term from the 1st concept area of geospatial modeling of environmental conditions coupled with each individual search term from the 2nd concept area of emerging infectious disease and scientific fields, which resulted in 40 searches in each of the five databases and in each of the seven journals for a total of 480 searches. Our search included articles published between 1995 and 2020 with no geographic or language limitations. Articles were included if they contained the specific terms shown in Table 1 that are associated with the concepts of geospatial modeling of environmental conditions and EIDs and scientific fields. Gray literature and articles reporting ENMs of species only and not involving an emerging infectious disease were excluded from our search. We managed citation, abstract, and article retrieval using Zotero (28).

**Table 1:** ENM features by studies in the systematic review of ecological niche modeling applied to vector-borne and zoonotic infectious diseases emergence.

Step	Prominent ENM features	Description	% Of studies
1	**Data approach**	Presence-absence	2%
		Presence-background	84%
		Presence-only	14%
	**Predictor selection**		
		Vegetation	36%
2	Environmental	Land cover types	43%
		Climate (temperature, precipitation)	89%
		Soil	21%
	Human social	Primarily population density and land use	13%
	**Algorithm applied**		
3	Machine learning	Maxent	55%
		GARP	22%
	Statistical algorithm	Generalized linear models, boosted regression tress	3%
	Mixed Methods	Maxent or GARP and boosted regression trees	20%
	**Model selection**		
4	Jackknife	Commonly used with maxent algorithm	38%
	Best Subset	Commonly used with GARP algorithm	23%
	Other	Chi-squared, bootstrap, etc. used with statistical algorithms	39%
5	**Model validation**	Area under the curve (AUC-ROC)	56%
		Partial area under the curve (pAUC)	13%
		Other	31%
6	**Uncertainty estimation**	Sensitivity analysis	7%

### 2.2. Article filtering

**Figure 2:**
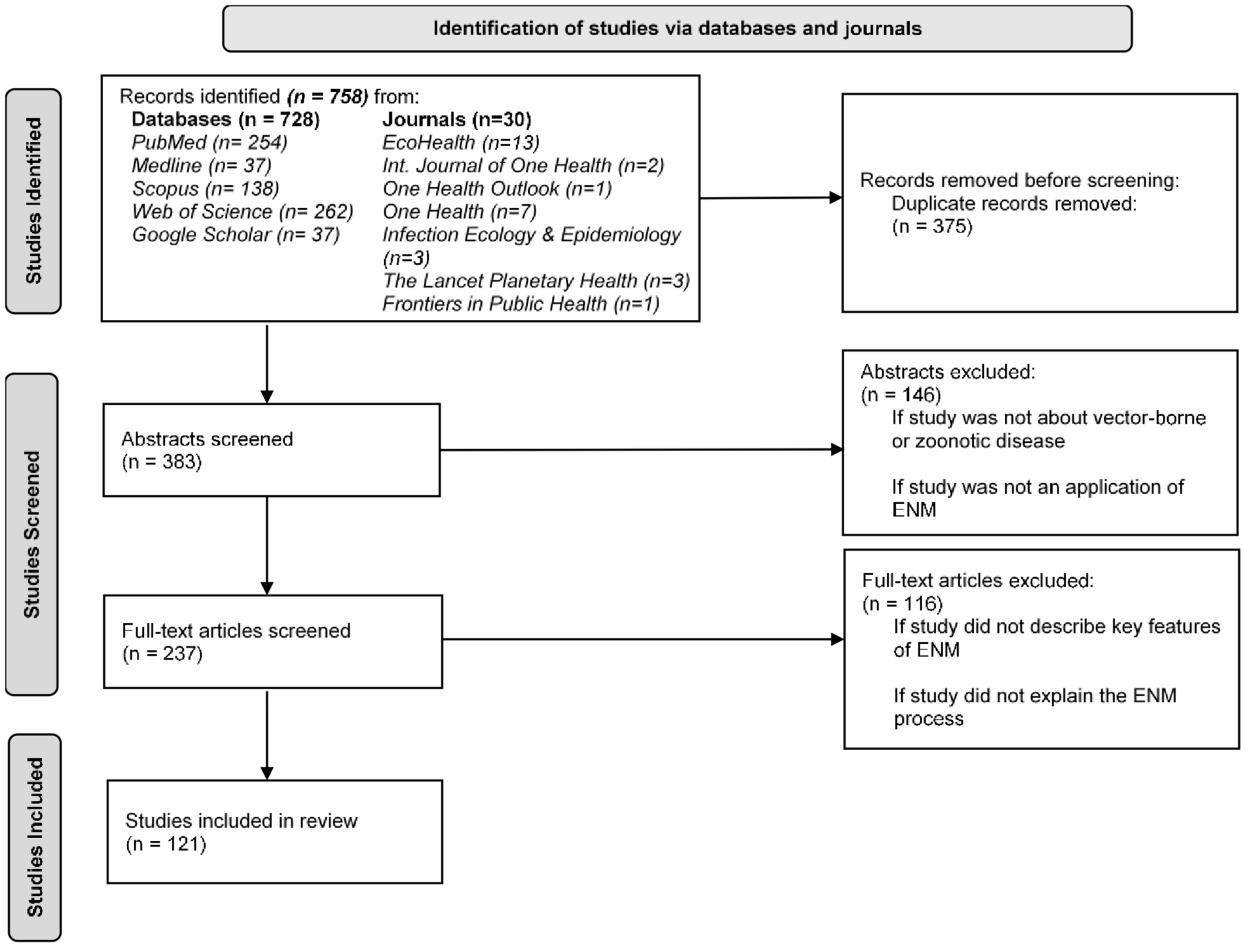
PRISMA flow diagram of studies identified, screened, and included in the systematic review of ecological niche modeling applied to vector-borne and zoonotic infectious diseases emergence.

Our search strategy resulted in a total 758 potentially relevant papers, and 383 articles after duplicates were removed (Figure 2) (29). Two reviewers screened all retrieved articles at the abstract and full-text levels. Articles were excluded (*n* = 146) at the abstract level if they: (i) did not examine a vector-borne or zoonotic disease; and (ii) were purely conceptual (i.e., review, perspective, and commentary) and had not included ENM. Thus, only articles that reported applications of ENMs of vector-borne or zoonotic infectious diseases, or those whose content was unclear based on reading the abstract alone were retained for the second step of the review. Next, the full-text articles were assessed, and articles were excluded (*n* = 116) if they: (i) did not describe key features of the ENM (i.e., describe and provide a rationale for data, algorithm, and application of the algorithm to data); and (ii) if they did not clearly explain or adequately describe the data, algorithm, application of the algorithm to the data, and the critical decisions necessary in the ENM construction process. Therefore, full-text articles were assessed to distinguish between studies that involved a click-and-run approach where researchers did not carefully consider and justify the data, algorithm, and application of the algorithm to the data (i.e., model construction process) versus studies that involved a robust-and-thorough approach that included some of the critical decisions necessary to develop a comprehensive, replicable ecological niche model that accounts for the natural history of the organism, the quality of the data, and the goal of the model (30). We based our full-text review criteria on Escobar (11) to differentiate between studies that did and did not adequately explain the ENM process and to identify studies that properly applied ENM to EIDs. Article exclusion rates for both reviewers at the abstract level were 63 and 55% and at the full-text level were 62 and 80%. The reviewers disagreed on 10% of articles at the abstract level and 33% at the full-text level. Disagreement between reviewers on potentially excluded articles were resolved by a blinded third reviewer and majority vote. In total, 121 publications were retained for analysis.

### 2.3. Data compilation, analysis and synthesis

Based on the studies that we identified as properly applying ENM to EID, we compiled and categorized data from the articles into a database according to the table in Appendix 2. First, we assessed publications over time, journal field, geographic distribution of study location by country, and disease characteristics and emergence factors with the studies. Next, we geolocated and mapped the studies by country and according to each study’s selection of social and environmental predictor variables, particularly climate zones, land cover type, and land-use biomes. We then assessed the studies according to integration of biotic, abiotic, and mobility factors. In particular, we evaluated the effectiveness of the studies to represent ecological niches based on the BAM framework (13, 21–23, 31). The evaluation comprises the identification of biotic, abiotic, and mobility factors included in the study, and specifically the combination of variables included (e.g., abiotic and mobility or biotic and mobility), which may represent potential versus actual ecological niches and sink or dead-end host populations versus source host populations, and the extent to which the geographic regions of the biotic, abiotic, and mobility factors overlap. Additionally, the ENM features were extracted from the articles and collated according to the criteria shown in Appendix 3. We divided the methodological framework into six steps according to methodological approach (presence-absence, presence-background, and presence-only), data selection, choice and application of algorithm, model selection, model validation, and uncertainty estimation. We describe the ENM features according to the percent of articles that exhibit specific features, number of articles with the most common features, and a description of the most common features included and excluded. We integrated the collated information into a stepwise ENM process and provide a descriptive summary of the essential details that need to be reported in published articles that apply ENM to EIDs.

## 3. Results

**Figure 3:**
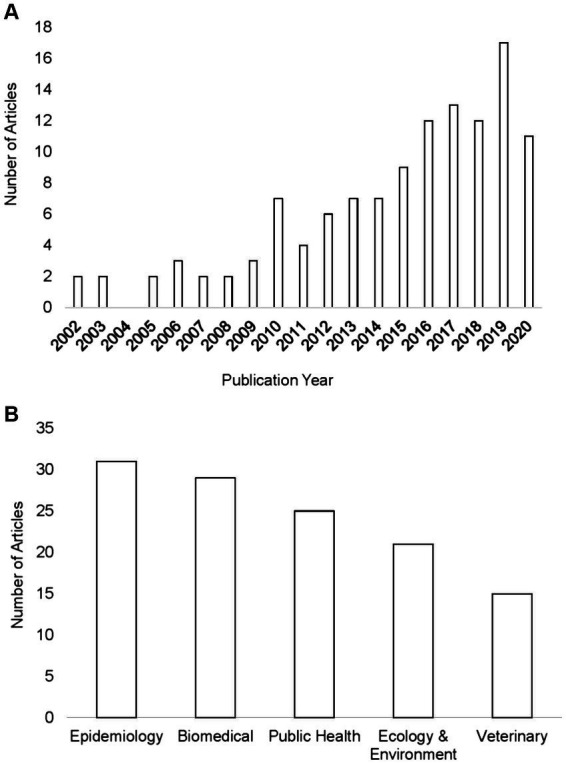
Number of publications in the systematic review shown by year. Our review had no articles prior to 2002 or in 2004 that met our criteria with a total of 121 articles between 2002 and 2020 **(A)**. Number of studies in the systematic review of ecological niche modeling applied to vector-borne and zoonotic infectious diseases emergence shown according to field of the journal **(B)**.

**Figure 4:**
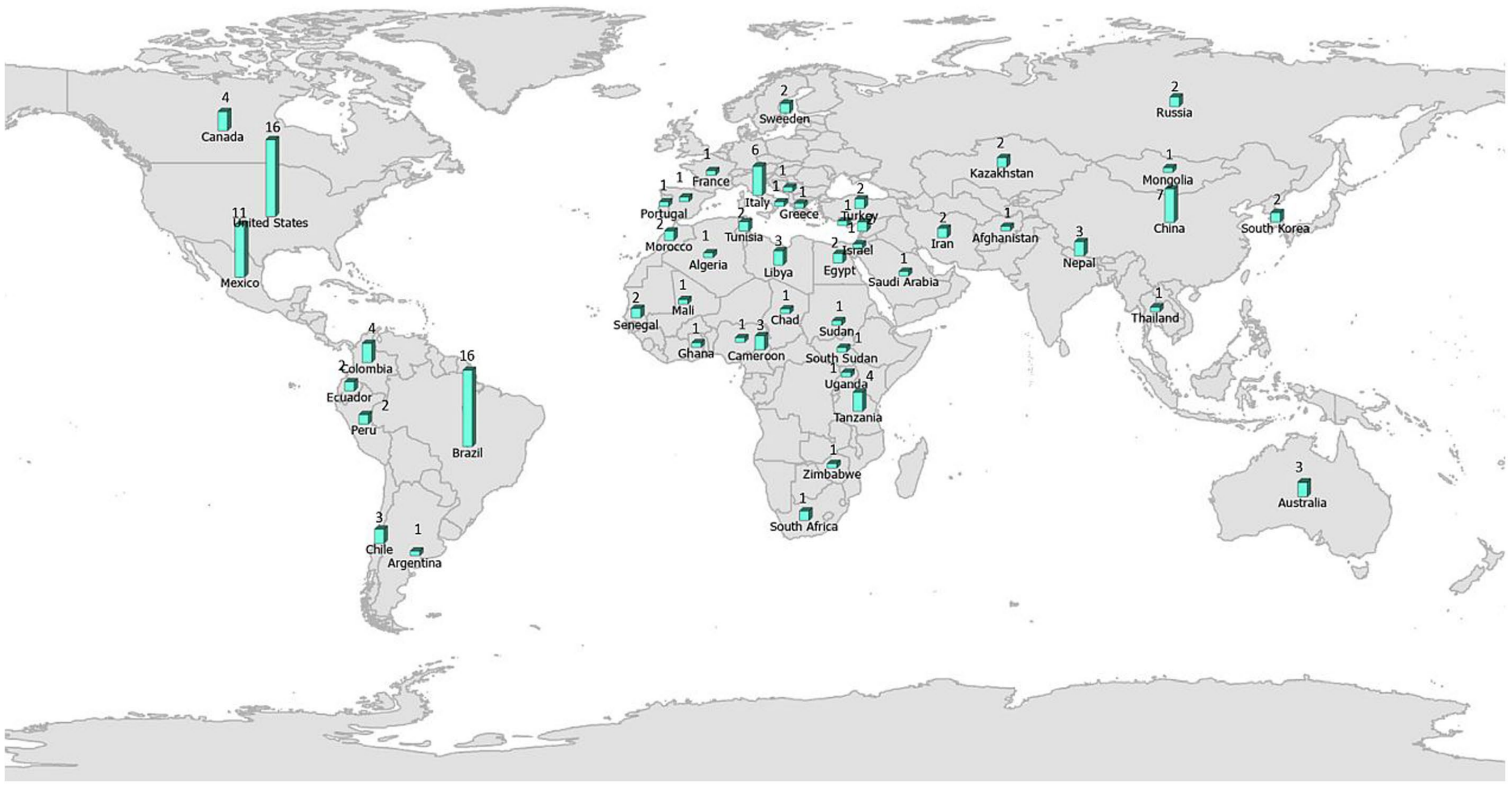
All (121) studies in the systematic review of ecological niche modeling applied to vector-borne and zoonotic infectious diseases emergence mapped by country. Map source: https://www.arcgis.com/home/item.html?id=deb60dd7744048cd9ba4fe203881fd12.

**Figure 5:**
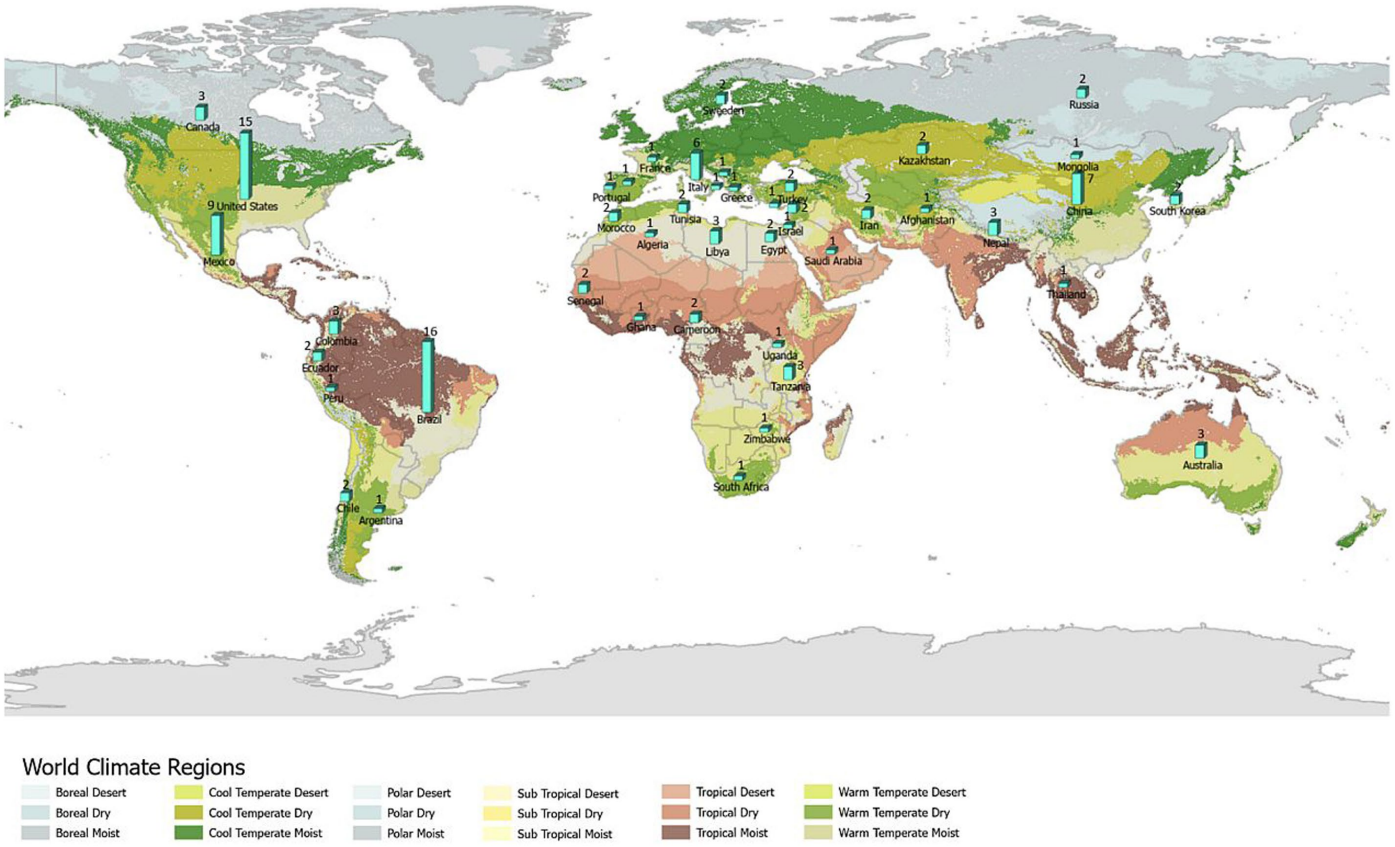
All (108) studies in the systematic review of ecological niche modeling applied to vector-borne and zoonotic infectious diseases emergence using climate variables in the ENM and mapped by global climate zone. Map source: https://storymaps.arcgis.com/stories/61a5d4e9494f46c2b520a984b2398f3b.

**Figure 6:**
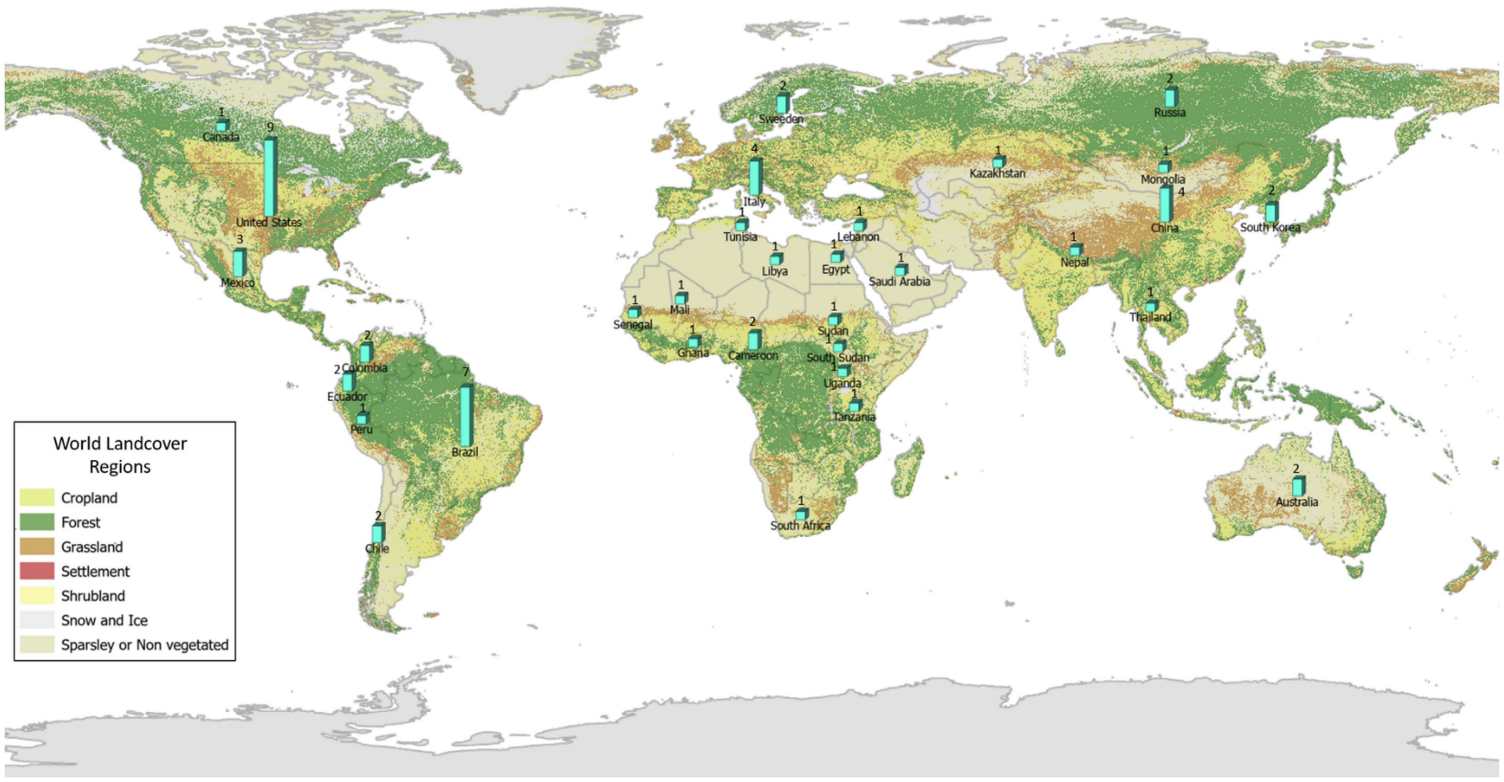
All (63) studies in the systematic review of ecological niche modeling applied to vector-borne and zoonotic infectious diseases emergence using vegetation or land cover variables in ENM and mapped by global land cover regions. Map source: https://land.copernicus.eu/global/products/lc.

The number of studies that applied ENM to predict vector-borne and zoonotic diseases (*n* = 121) has steadily increased primarily over the last decade of this review, with only 2 or 3 studies per year in each year 2000–2009 (Figure 3A). Most studies were conducted in the fields of epidemiology (*n* = 31), biomedicine (*n* = 29), and public health (*n* = 25), whereas less than half as many were conducted in the fields of ecology and environment (*n* = 21) and veterinary medicine (*n* = 15; Figure 3B). The application of ENM to EIDs has been somewhat unevenly distributed geographically across North America (*n* = 27), South America (*n* = 29), Africa (*n* = 22), Asia (*n* = 22), Australia (*n* = 3), Europe (*n* = 10), as shown in Figure 4. Calculating the number of studies relative to the area of the regions showed that North America, South America, Africa, and Europe had 2–4 times more studies per million km^2^ than Australia and Asia. A majority of the studies (62%) were conducted in developing countries, while far fewer studies (33%) were conducted in developed countries (Country status was based on the UN World Economic Situation and Prospects Report, 2021). In terms of climate zones, the largest percent of studies (33%) were located in the tropical climate zone compared to 22% in the subtropics, 21% in warm temperate, 19% in cool temperate, and 4% in polar climate zones (*n* = 108; Figure 5). A similar distribution exists across global land cover regions, but there were far fewer studies that consider the biotic conditions, such as vegetation or land cover (*n* = 65; Figure 6).

**Figure 7:**
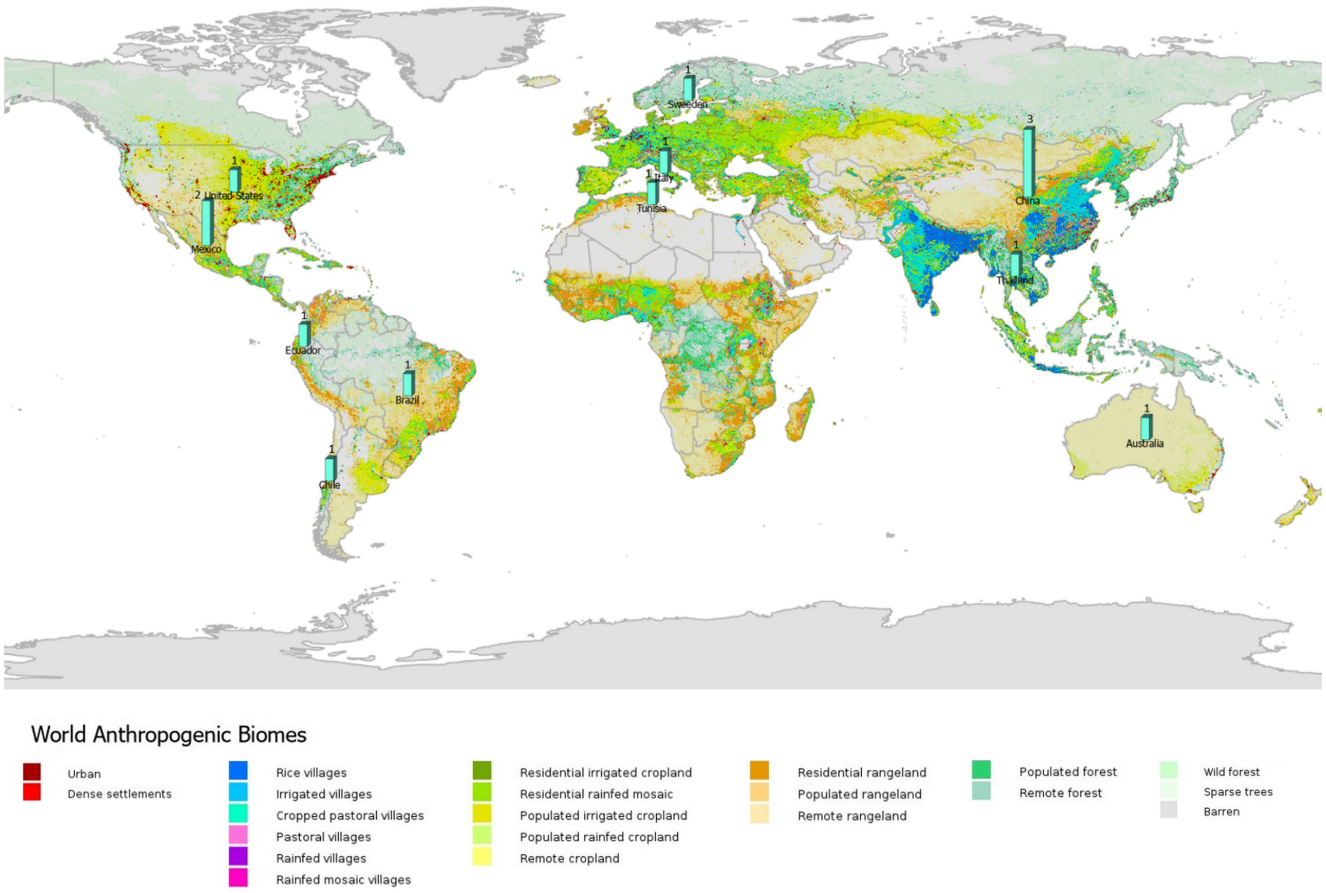
All (14) studies in the systematic review of ecological niche modeling applied to vector-borne and zoonotic infectious diseases emergence using social variables (e.g., land use) in ENM and mapped by anthropogenic biomes. Map source: https://sedac.ciesin.columbia.edu/data/collection/anthromes.

**Figure 8:**
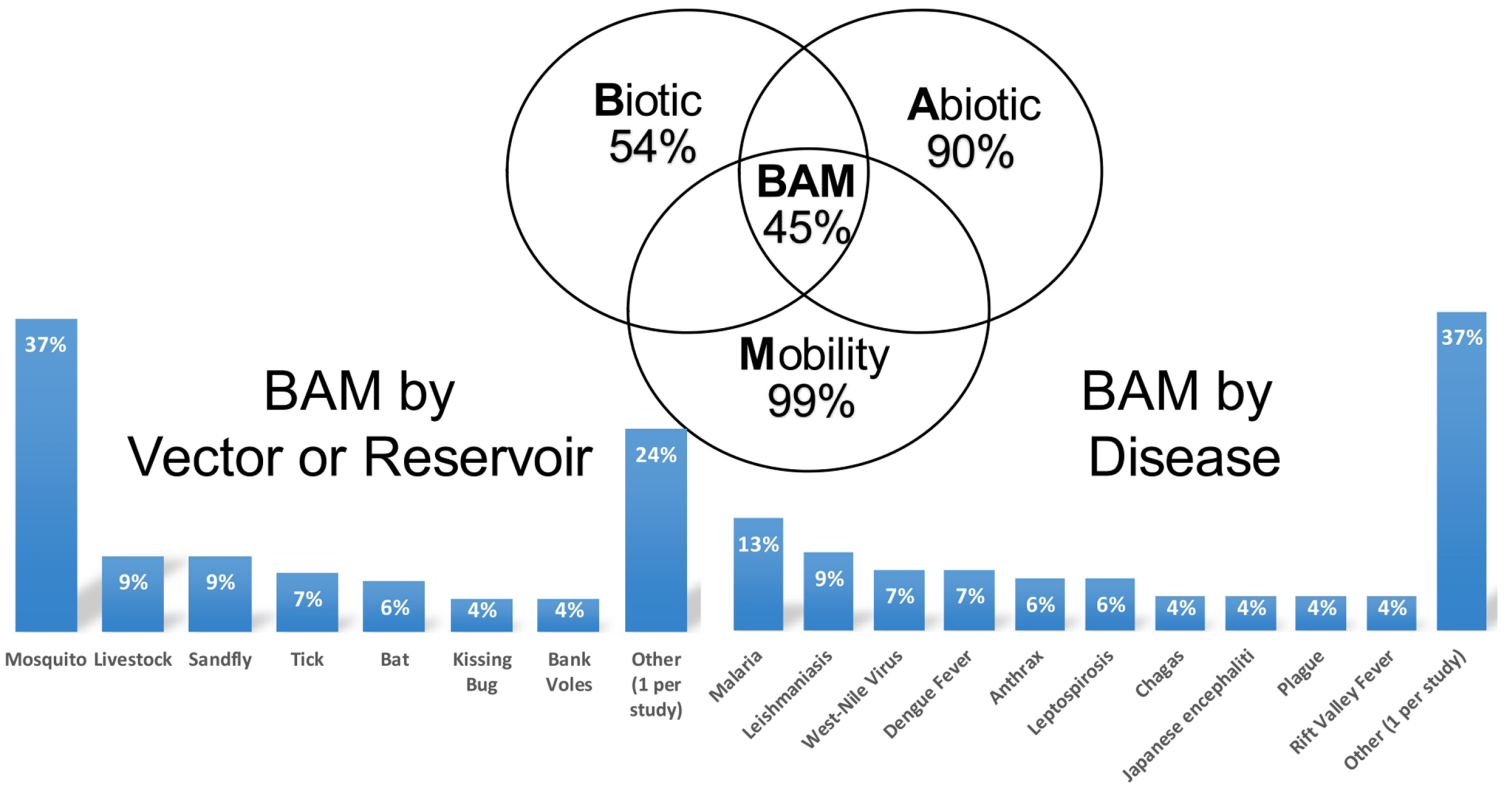
BAM framework applied in studies in the systematic review of ecological niche modeling applied to vector-borne and zoonotic infectious disease emergence. In all of the studies included in the systematic review, most examined abiotic factors (e.g., physical environment) and mobility factors (e.g., barriers to species’ dispersal ability). Fewer studies included biotic conditions (e.g., vegetation and land cover) and interactions (e.g., host species density), as well as included all (biotic, abiotic, mobility) factors consistent with the BAM framework. The BAM framework was mostly applied to mosquitoes followed by sandflies and livestock as vectors or reservoirs, and Malaria, Leishmaniasis, West Nile virus, and Dengue as diseases. Other vectors/reservoirs included midge, mite, rodent, puma, tape worm, water bug, and horses, while the other diseases included Zika virus, Chikungunya, Alveolar echinococcosis, Brucellosis, Hantavirus, Hendra virus, Mycetoma, Nipah virus, Piroplasmosis, Puumala virus, Pythiosis, Rabies, Thrombocytopenia, and African horse sickness.

Very few studies accounted for the anthropogenic factors (e.g., land use and resource overexploitation) that modify the spatial distribution of EIDs (*n* = 14; Figure 7). Of the studies that included social modify factors, all included human population data and to a lesser extent (roughly 1/3) included land-use data. The studies primarily used abiotic factors (e.g., physical environment; 90%; *n* = 109) and mobility (e.g., species’ dispersal ability; 99%; *n* = 120) in the application of ENM to predict vector-borne and zoonotic diseases (Figure 8). Only 54% of studies included biotic conditions and interactions (*n* = 65). Additionally, few studies (7%, *n* = 9) included only biotic and mobility factors, while 45% of studies modeled only abiotic and mobility factors (*n* = 54) and similarly (45%; *n* = 55) studies included all (biotic, abiotic, mobility) factors consistent with the BAM framework. The BAM framework was mostly applied to mosquitoes followed by sandflies and livestock as vectors or reservoirs, and Malaria, Leishmaniasis, West Nile virus, and Dengue as diseases, as shown in Figure 8.

A large majority of studies involve a presence-background approach (*n* = 102) with few studies using presence-only (*n* = 17) and presence-absence (*n* = 2) approaches, as shown in Table 1. Most studies focus on environmental predictors rather than modifying social factors. Specifically, climate variables are most often used (89%; *n* = 108) as predictors of disease emergence followed by land cover (43%; *n* = 52), vegetation cover (36%; *n* = 44), and soil characteristics (21%; *n* = 25). However, host species density alone was included in nearly all studies (96%; *n* = 116). Few studies (13%; *n* = 16) include human social variables as modifying factors in ENM to predict vector-borne and zoonotic diseases. The studies often utilized machine learning (88%; *n* = 106). Particularly, the MaxEnt algorithm was used in 64% of studies (*n* = 77). The GARP algorithm (Genetic Algorithm for Rule-set Production) was applied in 25% of the studies (*n* = 30). Most studies (74%; *n* = 90) reported model selection procedures. The Jackknife procedure, where each variable is excluded in turn to test model performance and a model is created with the remaining variables, was commonly used with MaxEnt. The Best Subset procedure (all possible models are compared using a specified data-driven set of predictors) was commonly used with GARP for model selection. Most studies (90%; *n* = 109) reported model validation procedures with most studies applying the Area Under the Curve (AUC) of the Receiver Operator Characteristic (ROC), which provides an aggregate measure of performance across all possible classification thresholds. The next most common validation procedures were a binomial probability approach and the true skill statistic. Only 7% of studies (*n* = 8) reported uncertainty analysis.

## 4. Discussion

### 4.1. Growth of ENM applied to EIDs and the distribution of studies

The uneven distribution of ENM to predict vector-borne and zoonotic diseases across journal fields in the past 20 years indicates that an increased engagement with the environmental and veterinary sciences is needed. Such engagement can result in greater insights into data selection, such as biotic factors contributing to emergence (26). Further, there was uneven geographic distribution of studies throughout the world. Most studies were in developing countries (62%), which is likely because most studied diseases are found in higher rates and with more negative impacts in developing regions. Also, most studies (33%) were within tropical climate zones where many infectious diseases emerge, which reflects the need to conduct studies in locations that are most vulnerable to emergence, and which likely comprises less-developed tropical countries with fewer resources and health infrastructure to respond to outbreaks. Nevertheless, continuing the growth and utility of spatial data and analyses will enable better predictions of EIDs and much-needed interventions earlier.

### 4.2. Likely effectiveness of reviewed studies to represent ecological niches

Studies that focus solely on abiotic and mobility factors (45%) comprise regions in geographic space where conditions allow growth rates of host-species populations to be positive, but the physiological conditions limit the ability of a host species to persist in that area (13, 32). As a result, such studies likely have investigated host-species populations where the infectious disease was (at least temporarily) not found or was potentially transient in dead-end hosts, which limit the persistence of an infectious disease (22). Essentially, the combination of only abiotic and mobility factors can represent a sink of host-species populations where host species are unable to persist (13). Similarly, the studies that focused solely on biotic and mobility factors (7%) also represent potential sink populations where an infectious disease is (at least temporarily) not found or potentially transient in dead-end hosts (22). For example, predation and competition between species may restrict a host-species distributions and the infectious disease may predispose the host species to increased predation. Stable host-species populations will be found primarily in regions where the ENM includes biotic, abiotic and mobility factors (13). Such a region has the appropriate set of biotic and abiotic factors for the host species to be present and persist, is accessible to the host species, and is equivalent to the geographic distribution of the host species (32).

### 4.3. Potential pitfalls of ENM techniques in reviewed studies

The popularity of the MaxEnt (Maximum Entropy) algorithm and the Genetic Algorithm for Rule-set Prediction (GARP) is apparent in our systematic review. Despite the heavy reliance on machine learning techniques, other statistical algorithms were used in a few studies of our review to predict EIDs, such as generalized linear models and boosted regression trees. Notably, boosted regression trees were used in conjunction with MaxEnt or GARP in a sizable number (20%) of studies in our review. Still, the application of the MaxEnt or GARP algorithms can present different challenges (21, 33, 34). In particular, the predictive performance may vary depending on the modeled host species’ ecological characteristics as the different properties of the data and/or of the species may affect the accuracy of predictive maps (35). When applying ENM to infectious diseases users should use multiple metrics to assess how the data and analyses influence predictive performance. Further, model selection procedures chosen in the studies were often associated with the algorithm applied to the data and differ in important ways. When applying ENM it is important to understand that the Jackknife test is primarily used in the model development process to assess the contribution of each variable to the model (36, 37). The jackknife test runs the model (1) once with all variables, (2) dropping out each variable in turn, and (3) with a single variable at a time (36). The importance of a variable is determined based on having a large training gain when the variable is used alone in the model and a subsequent decrease in training gain when removed from the model (38). Alternatively, the Best Subset procedure assess multiple possible models and it presents the best candidates, but it is up to the user to compare and choose one model (39). Further, the best model is not always apparent, and judgment is required.

Model performance evaluated in studies that were included in our review primarily used the area under the curve (AUC) of the receiver operating characteristic (ROC). However, using such an approach has been criticized (40, 41). Particularly, Lobo et al. (42) recommend not using AUC for five reasons: (1) it ignores the predicted probability values and the goodness-of-fit of the model; (2) it summarizes the test performance over regions of the ROC space in which one would rarely operate; (3) it weights omission and commission errors equally; (4) it does not give information about the spatial distribution of model errors; and, most importantly, (5) the total extent to which models are carried out highly influences the rate of well-predicted absences and the AUC scores. To account for the potential limitations of the AUC in 15 of the reviewed articles published between 2015 and 2020, the overall accuracy of the models was assessed using the partial AUC (pAUC) procedure (43), which allows the user to set bounds on the types of predictions that are to be considered when certain portions of that space are not directly relevant to applications of interest. Overall, pAUC seems recently considered as more practically relevant than AUC (44).

Few studies conducted uncertainty analysis. When ENMs are transferred in space and time, it is important to understand the sources and location of uncertainty in their predictions (45). Uncertainties associated with ENMs have increased partially because of the increased number of environmental datasets available (46). Measuring uncertainty is crucial when applying ENM to predict EIDs as uncertainty can arise from several sources (45). Some studies in our review did test whether uncertainties were related to the choice of methods. Still, more uncertainty analyses are needed in future studies.

### 4.4. Recommendations for future studies

Despite the importance of anthropogenic pressures to EIDs few studies (roughly 10%) that we assessed included such variables in ENM. Further, all of the studies primarily used human population growth or density as a predictor that modifies the emergence and spread of infectious diseases. Far more attention must be given to a wide range of human social factors that modify the behavior and presence of a host species in future ENM studies. Land use is another important variable that was used in only a few of those small number of studies that included social (modifying) factors. Such factors that modify or influence the behavior and presence of a host species is particularly important to include in ENM when modeling domesticated vs. wild host species, such as livestock. Additionally, other modeling approaches, such as agent-based modeling and metapopulation models are used in epidemiology but overlooked in ENM and could be utilized to contribute to the inclusion of social factors. Agent-based models require detailed data entry for each individual in the population of interest resulting in a comprehensive description of the human behavior linked to an epidemic, while structured metapopulation models take into account geographic area census data and interpopulation mobility patterns (47). Overall, the lack of social factors (e.g., land use, land tenure, human population density) that can modify disease patterns reflects an overly simplistic view of pathogens, and disconnects pathogens from social and ecological contexts, whereas a holistic perspective that incorporates social as well as physical, chemical, and biological dimensions of our planet’s systems is more realistic (7).

Biotic conditions and interactions are closely linked to landscape structure (48). Specifically, biotic factors, such as species richness, distribution, population density and the structure of host communities and reservoirs are essential in the transmission of infectious or parasitic agents (49, 50). Population and community structure are further associated with habitat variables, such as the amount of habitat available for species in a single land cover patch or the total amount of habitat available for species across all the different land cover patches, which can influence the type, frequency, and intensity of biotic interactions (48). Generally, habitat is delineated and included in ENM through vegetation and land-cover imagery (51), which were applied in most (95%) of studies that modeled all BAM components. In turn, changes to habitat structure (e.g., fragmentation) affects the prevalence of infectious diseases (50). However, our systematic review revealed a lack of studies overall that include biotic factors in ENM. The lack of inclusion may be because biotic conditions and interactions are hypothesized to affect distributions only locally, but there is growing evidence that biotic interactions may have a larger role in shaping broad-scale distributions (22). Thus, more effort is needed to test and include biotic variables in ENM, such as the number of different habitat types, the prevalence of edge habitat, and the configuration of habitat. Habitat edges in particular can influence biotic interactions through forming a barrier to species movement and dispersal, may have differential influences on species’ populations and change the intensity of outcomes of biotic interactions, and the proximity to edge and the associated influence of surrounding matrix can also affect the outcome of biotic interactions (48).

Future studies should consider the full BAM approach in developing ENM of EIDS. Furthermore, the spatial overlap of the BAM components must be carefully considered as an ENM study can result in estimating the potential rather than the actual host-species niche (13). Our review indicates most studies tend to focus on abiotic conditions and mobility factors. Even when biotic factors are included together with abiotic and mobility factors, the ENM can still represent the geographic space of primarily the abiotic and mobility factors. In such a case, the biotic interactions restrict the presence of host-species populations to a small spatial extent of geographic space of the abiotic and mobility factors (21). Additionally, the ENM can represent the geographic space of primarily the biotic and abiotic factors. Even if regions of biotic and abiotic largely overlap, the interactions can be weak, diffuse and non-specific if the accessible region due to mobility factors overlap with the regions of biotic and abiotic in only a relatively small region (13). In such a situation the potential niche of the host-species population will generally be larger than the actual distribution of the host-species population’s actual niche. Overall, unless the three sets of factors affecting geographic distributions of species overlap almost entirely, most ENM algorithms estimate the potential rather than the actual niche. These considerations are foremost if ENMs are to have biological meaning and reality (52).

## 5. Conclusion

The use of ENM to predict EIDs is growing, and our review can help guide this approach. We clarified the socio-environmental factors that are commonly implicated as drivers of vector-borne and zoonotic diseases, social and environmental settings of study locations, the variables modeled, and the principal elements of ENM and the modeling process to predict EIDs. Rigorous modeling of underlying drivers of EIDs can better inform epidemiological and One Health professionals to help prevent outbreaks and future epidemics.

## Data availability statement

The raw data supporting the conclusions of this article will be made available by the authors, without undue reservation.

## Author contributions

TL conceptualized the systematic review, was the third reviewer to settle disagreements on articles through majority voting during the abstract and full-text screening, conducted the data analyses, verified the underlying data, and was the primary author of the article. BT conducted article searches in the research databases, abstract and full-text screening, verified the underlying data, and managed the systematic review database and workflow processes. AG conducted article searches in the research databases, abstract, and full-text screening. DT provided guidance and validation of the systematic review methods. SD, EF, IG, VS, and ES had full access to the data, contributed equally to the interpretation of data, and critically revising the manuscript for important intellectual content. All authors contributed to the article and approved the submitted version.
