# Supplementary material for: A global examination of ecological niche modeling to predict emerging infectious diseases: a systematic review
Source: Front Public Health. 2023 Nov 2;11:1244084. doi: 10.3389/fpubh.2023.1244084 (PMC10652780; doi:10.3389/fpubh.2023.1244084)
Supplement: Supplementary file 1 [file Data_Sheet_1.docx]

**Appendix 1** Search terms used to retrieve articles for systematic review of ecological niche modeling applied to vector-borne and zoonotic infectious disease emergence.

**Appendix 2** Study information compiled per article in the systematic review of ecological niche modeling applied to vector-borne and zoonotic infectious diseases emergence.

**Appendix 3** ENM features compiled from articles in the systematic review of ENM applied to vector-borne and zoonotic infectious diseases emergence.
